# In This Issue: Volume 117, Issue 2, February 2026

**DOI:** 10.1111/cas.70321

**Published:** 2026-02-01

## Stromal Defense Against Cancer: An Unprecedented Mechanism Limiting Cancer Invasion Into the Bone

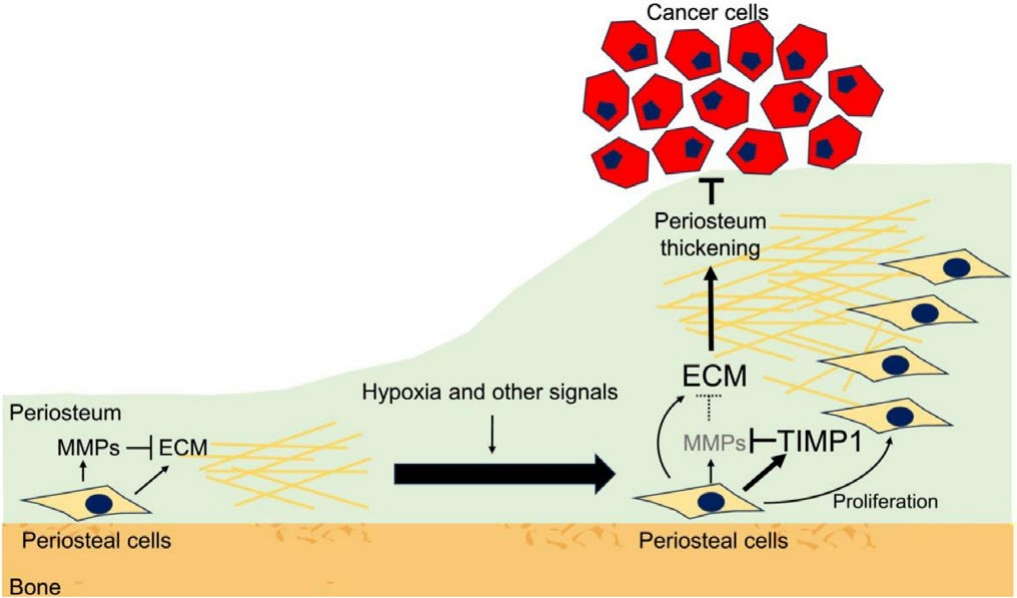

Head and neck squamous cell carcinoma (HNSCC) is a type of cancer arising from epithelial cells that line the oral and respiratory tracts. As HNSCC progresses, cancer cells often invade adjacent jawbones, which significantly worsens patient prognosis and quality of life. Although several factors contributing to bone invasion have been identified, the mechanisms regulating how tumors interact with bone‐associated tissues have not been fully understood. In a recent review, Tsukasaki M and Takayanagi H summarized the molecular and cellular mechanisms underlying bone invasion in HNSCC.

The authors focused on the role of the periosteum—a membrane that covers the outer surface of the bone—in the bone invasion of HNSCC.

Using an HNSCC mouse model, they found that tumors implanted near bone without physical periosteal damage led to significant periosteal thickening. Normally, periosteal stromal cells maintain periosteal thickness through balanced production of extracellular matrix factors and metalloproteinases. However, the low oxygen conditions (hypoxia) of the tumor microenvironment activate hypoxia‐related signaling (HIF‐1α signaling) in periosteal stromal cells, driving TIMP1 (an inhibitor of protein‐degrading enzymes) production. These alterations result in periosteal thickening. This thickened periosteum serves as a physical barrier, preventing cancer cell invasion into the bone.

Periosteal thickening is followed by new bone formation in cases of some infectious diseases or injuries but not in HNSCC. This may be because the dividing HNSCC cells and accumulating myeloid cells produce protein‐degrading enzymes to a level that exceeds the inhibitory capacity of periosteal TIMP1. Therefore, the protective physical barrier of the thickened periosteum is breached, allowing the HNSCC cells to invade the bone. The bone invasion of cancer cells decreases periosteal stromal cell number, HIF‐1α signaling, and TIMP1 production, preventing the formation of the protective barrier and further driving cancer cell invasion.

These findings can be harnessed for developing a novel treatment strategy for HNSCC by preventing bone invasion. This strategy is distinct from the traditional cancer treatment strategy of targeting cancer cells or their microenvironment.

https://onlinelibrary.wiley.com/doi/full/10.1111/cas.70273

## Regulation of R‐Loop Dynamics by Proteins and Long Noncoding RNAs: An Emerging Paradigm for Cancer Treatment

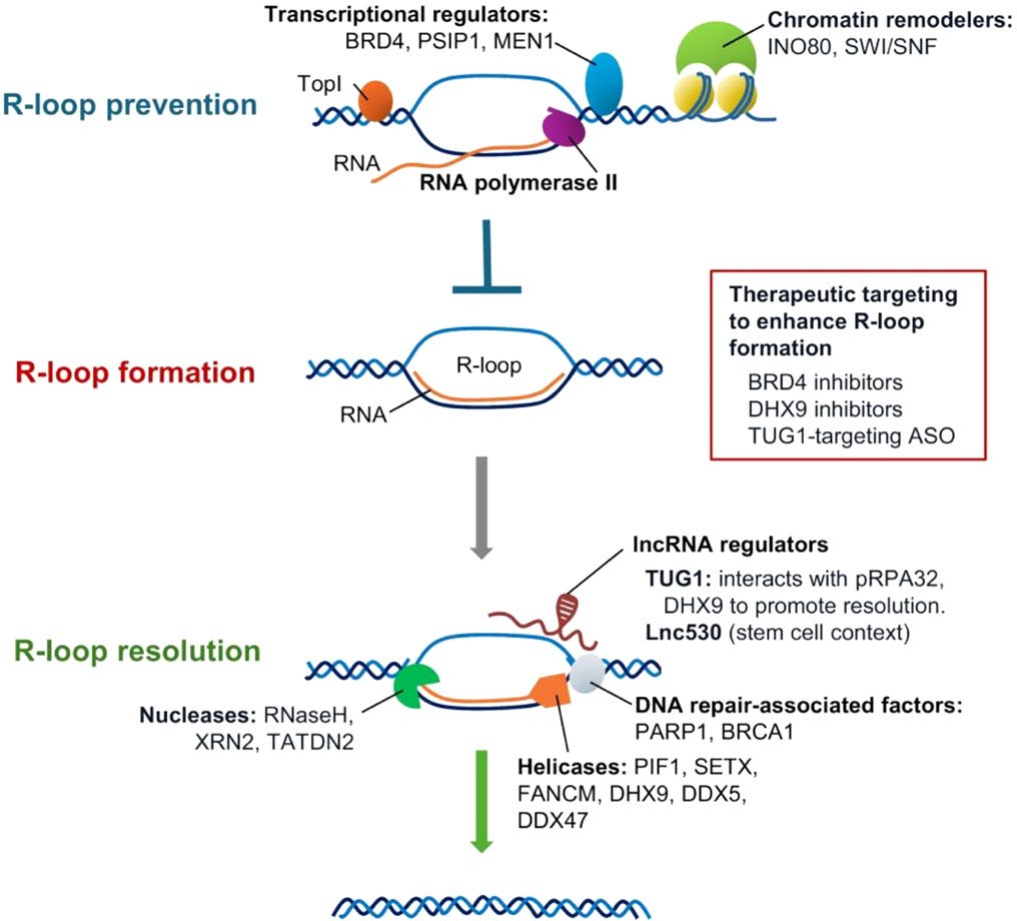

Cancer cells grow and divide rapidly, placing enormous strain on their genetic material. One major source of this stress arises from structures called R‐loops, which form when newly made RNA binds back to its template DNA strand during transcription. While short‐lived R‐loops are a normal part of gene regulation, their accumulation can damage DNA, disrupt replication, and drive genome instability. This creates a strong dependence on the pathways that regulate R‐loops.

This review brings together recent research to explain how cancer cells control R‐loops using a coordinated set of proteins. Some proteins help package DNA, while others remove harmful RNA–DNA structures. Together, these systems limit R‐loop build‐up and protect DNA. A key new finding is that long noncoding RNAs also help regulate R‐loops. The RNA called TUG1 acts as a scaffold when DNA is under stress. This challenges the earlier view that only proteins control R‐loops and shows that RNA itself plays an active role in maintaining genome stability in cancer cells. The study also shows that R‐loops are not always harmful. This highlights the need for careful control of R‐loops rather than their complete removal.

Together, these findings show that R‐loop control is both a weakness and an opportunity in cancer. Cancer cells depend on specific proteins and RNAs to manage R‐loops and survive DNA stress. Targeting these molecules may overwhelm cancer cells while leaving healthy cells less affected. This approach could open new paths for more precise cancer treatments.

https://onlinelibrary.wiley.com/doi/full/10.1111/cas.70272

## From Genomic and Epigenomic Maps to Medicines in Adult T‐Cell Leukemia/Lymphoma

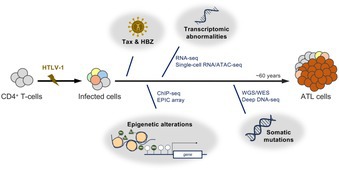

Adult T‐cell leukemia/lymphoma (ATL) is a rare cancer of immune cells called T cells. It is caused by infection with a virus known as human T‐cell leukemia virus type 1 (HTLV‐1). After the virus infects T cells, it inserts its genetic material into the cells. This changes how the cells behave and helps them grow and avoid the immune system. Over time, these infected cells also build up harmful changes in their genes. These changes develop slowly over many years during a long “quiet” period before symptoms appear. Together, long‐term viral infection and these gradual genetic changes increase the risk that ATL will develop.

In this review, Suzuki and Yamagishi describe the molecular changes involved in ATL and explain why they matter for patient care. Many of these changes affect the genes of infected T cells and show that the cells become genetically unstable over time. Not all infected T cells change in the same way. Different groups of cells develop depending on the types of changes they acquire. Some changes affect how strongly genes are switched on or off. Others affect how genes are regulated without changing the DNA itself. Studies show that these changes can begin long before cancer develops. As infected cells collect more changes over time, some begin to grow faster, form expanding cell populations, and eventually become cancerous.

Studying mutation patterns in ATL has important clinical implications. These patterns help identify which cell pathways are affected and may improve understanding of how the disease begins. The way the disease progresses depends on the types of molecular changes present. By combining mutation information with clinical data, doctors may better predict outcomes and assess disease risk in people who carry the virus but have no symptoms. This could allow closer monitoring and support future preventive approaches. People with ATL can also respond differently to treatments depending on their mutation profile. Understanding these differences can help guide treatment choices and support more personalized care.

https://onlinelibrary.wiley.com/doi/full/10.1111/cas.70245

